# Dr. Gardner Carpenter Clarke

**Published:** 1897-06

**Authors:** C. G. Stivers, Fred R. Mcbrien

**Affiliations:** Committee; Committee


Dr. Gardner Carpenter Clarke, who died March 22, 1897, was the first president of the Niagara Falls Academy of Medicine. At the regular monthly meeting of the Academy, held April 5, 1897, the following memorial was adopted and spread upon the minutes.

Whereas: In view of the great loss that we have sustained through the death of our honorary president, Gardner C. Clarke, M. D., and the still greater affliction sustained by those nearest and dearest to him, be it

*Resolved*, That we esteem it a privilege to extend our sincere sympathy to his family in their affliction, and further, that in mourning his death we feel that we regret the loss of one who was worthy of all the love and confidence that he commanded while in our midst, both on account of his skill as a physician and his nobility as a man; therefore be it

*Resolved*, That these resolutions form a part of the minutes of this meeting and that they be printed in the daily papers and that engrossed copies be sent to his family.

C. G. STIVERS, M. D.

FRED R. McBRIEN, M. D., *Committee.*

